# Fleas as Carriers of Infection

**Published:** 1898-11-19

**Authors:** 


### FLEAS AS CARRIERS OF INFECTION.

A large portion of the current number of the *Annales de l'Institut Pasteur* is occupied by a communication from Dr. P. Simond, the object of which is to show the manner in which plague is spread. Whether the conclusions arrived at should prove to be correct or not, we must express our admiration for the great care with which the inquiry has been carried out and the forcible and convincing manner in which the author has placed his conclusions before his readers. His contention may be summarised shortly as follows. Plague is a disease which especially affects men and rats and is carried from subject to subject, be it from man to man, from rat to man, or from man to rat, by means of fleas, and perhaps, so far as transport from man to man is concerned, by means of bugs. There is something very taking about this theory. It seems to explain to some extent the undoubted relation between the mortality among men and among rats, the special incidence of the disease among those who dwell in dirt and poverty, and the tendency of the infection to cling to certain houses, which is one of its most marked peculiarities, while if we look upon the matter from an epidemiological standpoint and accept the suggestion that the disease spreads with far greater virulence among rats than among humans, there is much in the hypothesis to explain the curious tendency shown by plague to die out and then to recur again after a given interval, when a new generation of rats has appeared on the scene, or when those which had fled have returned again to their old quarters.

From all antiquity it has been observed that a great mortality among rats has preceded and accompanied epidemics of plague among human beings, but it is only since the discovery of the specific microbe of plague, and the demonstration of the identity of the disease among rats and among men that it has been possible to establish a relation of cause and effect between them. This relation, however, has been far from generally accepted. A link in the chain was missing, and this link Dr. Simond professes to have discovered in the blood-sucking parasites by which the infection is carried from subject to subject.

Not only has it been noted that a great mortality among rats has often preceded an outbreak of plague among men, but it has again and again been noticed that among the early victims of the disease have been those employed in the grain warehouses and other places where the mortality among the rats had been the greatest. It has also been shown that the individuals who have touched the recently dead rats have often been the first to be attacked with bubonic plague. Nevertheless, there are facts innumerable to show that contact with those suffering or recently dead from plague, whether they be men or rats, is not sufficient to convey the infection. Some other factor is wanted to explain how the infection is carried from case to case, and it is here that the flea comes in. The healthy rat takes much care about its person and keeps itself free from fleas, but when it is attacked by disease it ceases so to do, and the animals which die of plague are constantly found swarming with these parasites, all of which become potential carriers of infection. Thus we see how it is that the disease clings to houses which are ill kept, are on the bare soil, are crowded with inhabitants, and are infested, on the one hand, with rats, by whom the infection is introduced from other houses, and, on the other, by fleas, by which it is carried from the rats to the inhabitants.

Dr. Simond concludes, then, that man and the rat are the principal agents in the transport of the disease; man carrying it over long distances, and setting up fresh foci of infection, while the rat disseminates it locally, and thus becomes the effective agent in the production of epidemics. The introduction of infected rats into a place, he says, is generally followed after a short interval by the development of plague among the inhabitants. But the introduction of plague-stricken men into a place does not necessarily produce an epidemic. For the production of such a result a concourse of favourable circumstances is required, most important among which would seem to be the spread of the infection among rats. But this dissemination among rats, the spread of the disease from rat to rat, from rat to man, and back again from man to rat, is due not to the directly infectious nature of the disease, but to the infection being conveyed from one to the other by parasites common to both. An actual puncture of the skin is necessary for the communication of the disease, and this puncture is made by a blood-sucking parasite. We need hardly say how important such a theory is from a public health point of view. As in so many other cases, we find the infection not simply jumping across from case to case, but reaching its new victim by a comparatively complicated route; and if we can but break the chain at any point we may have good hope of stopping the progress of the disease. With good tight drains, with well-built houses into which rats cannot gain access, and with cleanly personal habits, by which fleas and other biting insects are kept at bay, plague, if this theory is to be believed, may be kept at bay also. According to its rats, its fleas, its bugs, and the tightness of its drains each city shall be judged—and we wonder how Southwark and Bermondsey, and the regions of the docks about the Commercial Road will fare when this test is applied! At first sight it would seem that such a theory proves too much, and that if parasites are the carriers of the disease, nurses ought to suffer to a greater degree than has been the case. In regard to this, however, it is to be borne in mind that fleas are the parasites of man's clothes rather than of man himself, and that the natives when brought to hospital are but lightly clad, and as bearing on the question one must not forget that in colder climates, where more clothes are worn, all history shows that the attendants on the sick have suffered heavily from plague.

